# Integration of single‐cell and bulk RNA sequencing unravels metalloendopeptidase+ neutrophils as key inflammatory drivers in abdominal aortic aneurysm

**DOI:** 10.1002/ctm2.70121

**Published:** 2024-12-02

**Authors:** Lushun Yuan, Chuan Liang, Shuzhen Cheng, Jianliang Zhou, Song Chen

**Affiliations:** ^1^ Department of Vascular Surgery Intervention Center Shanghai General Hospital, Shanghai Jiao Tong University School of Medicine Shanghai China; ^2^ Department of Cardiovascular Surgery Zhongnan Hospital of Wuhan University Wuhan China; ^3^ Hubei Provincial Engineering Research Center of Minimally Invasive Cardiovascular Surgery Wuhan China; ^4^ Wuhan Clinical Research Center for Minimally Invasive Treatment of Structural Heart Disease Wuhan China; ^5^ School of Food Science and Technology National Engineering Research Center of Seafood Collaborative Innovation Center of Seafood Deep Processing Dalian Polytechnic University Dalian China

1

Dear Editor,

In this study, we comprehensively investigated the immune microenvironment of abdominal aortic aneurysm (AAA) and unveiled a novel neutrophil subtype as a key inflammatory driver.

AAA is a prevalent vascular condition characterized by significant mortality, primarily due to the risk of aortic rupture.^1^ Currently, there are no established pharmacological interventions to effectively mitigate AAA growth or prevent rupture. Previous research has found key pathological features linked to AAA, such as smooth muscle cell (SMC) depletion, elastin fragmentation and extracellular matrix degradation. These factors lead to impaired vascular remodelling and weaken the aortic wall.^2^, ^3^ Recently, increasing attention has been directed towards the role of immune cells in AAA pathogenesis.^4^, ^5^, ^6^, ^7^ Several investigations have utilized single‐cell RNA sequencing (scRNA‐seq) to explore the heterogeneity and cellular responses of multiple cell types during AAA development.^2^, ^8^, ^9^, ^10^ However, these studies predominantly employed mouse models, identified limited cell types and did not conduct in‐depth analyses on some specific cell types. Furthermore, the focus of these studies has largely been on SMCs and fibroblasts, leaving the characterization of the immune microenvironment in human aortic tissues underexplored. To bridge this gap, we utilized scRNA‐seq to comprehensively elucidate the immune microenvironment associated with AAA.

Initially, we conducted scRNA‐seq analysis on healthy aorta samples from three heart transplant recipients alongside AAA content from three patients with AAA (Table S1). A total of 20,190 qualified cells were used for further analysis (Figure 1A) and 11 distinct clusters were uncovered (Figure S1). By examining conserved genes within each cluster, we identified five major cell types: endothelial cells, SMCs, myeloid cells (including neutrophils and macrophages), a combined cluster of natural killer (NK) cells, NKT and T cells, B cells (including B cells and plasma cells) and proliferative cells. Notably, the immune microenvironment within AAA exhibited distinct characteristics compared to healthy aorta samples (Figure 1B–E). This analysis provided valuable insights into the cellular composition and immune landscape of AAA, highlighting the differences between AAA contents and healthy aorta.

**FIGURE 1:**
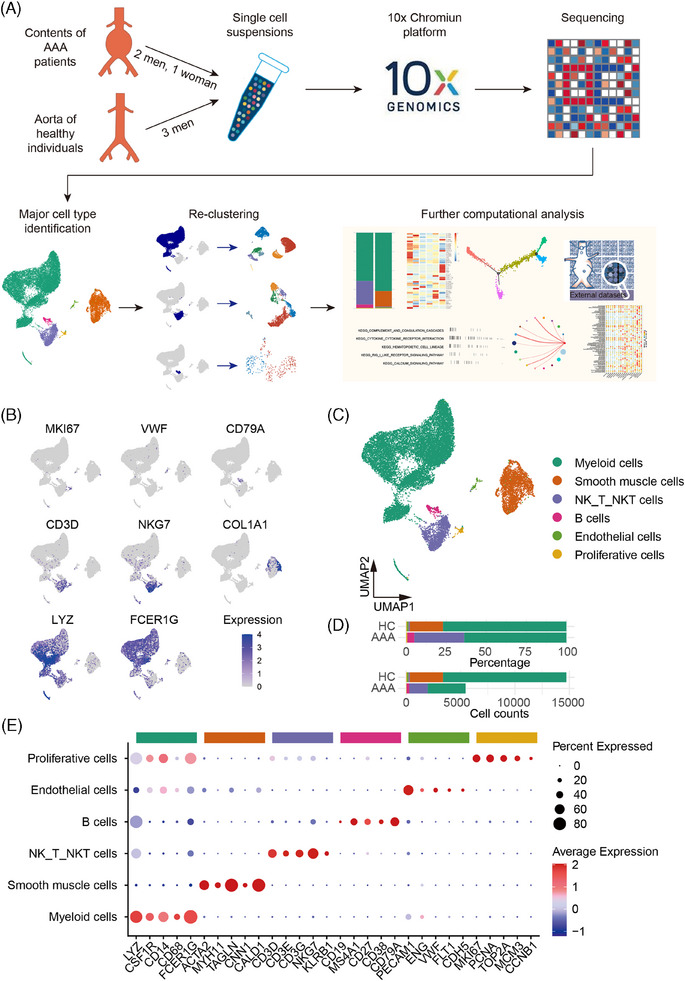
Five major cell types identified with single‐cell RNA sequencing analysis of contents of human abdominal aortic aneurysm and healthy aortic tissues. (A) Experimental design and data analysis strategy. (B) Relative expression of several marker genes. Cells were visualized on a UMAP plot. (C) UMAP plot showing all cells colour‐coded according to their respective major cell types. (D) Horizontal bar plot illustrating the composition and count of each cell type (composition calculated as the total number of control cells divided by the total number of cells across all specimens). (E) Mean expression of selected genes in the major cell types. *AAA* abdominal aortic aneurysm; *HC* healthy control; *UMAP* Uniform manifold approximation and projection.

Re‐clustering analysis of myeloid cells revealed seven distinct subpopulations: three resident macrophages exclusively found in the healthy aorta, and two inflammatory macrophage subtypes alongside two classes of inflammatory neutrophils within AAA (Figure 2A,B). Notably, hierarchical clustering analysis showed that inflammatory subpopulations, particularly the membrane metalloendopeptidase (MME)+ neutrophils, clustered closely with CCL5+ macrophages (Figure 2C), highlighting a potential interplay between these cell types. Subsequent analysis of functional signalling molecules and pathways revealed cell‐type‐specific patterns within the human leukocyte antigen system across these six subpopulations (Figure 2D). Resident and inflammatory macrophages express major histocompatibility complex (MHC) class II molecules but exhibit distinct features. A notable highlight is the role of MME+ neutrophils, which predominantly express MHC class I molecules. These inflammatory neutrophils are implicated in immune defence mechanisms and the release of cytotoxic compounds, potentially exacerbating the inflammatory processes linked to AAA.

**FIGURE 2:**
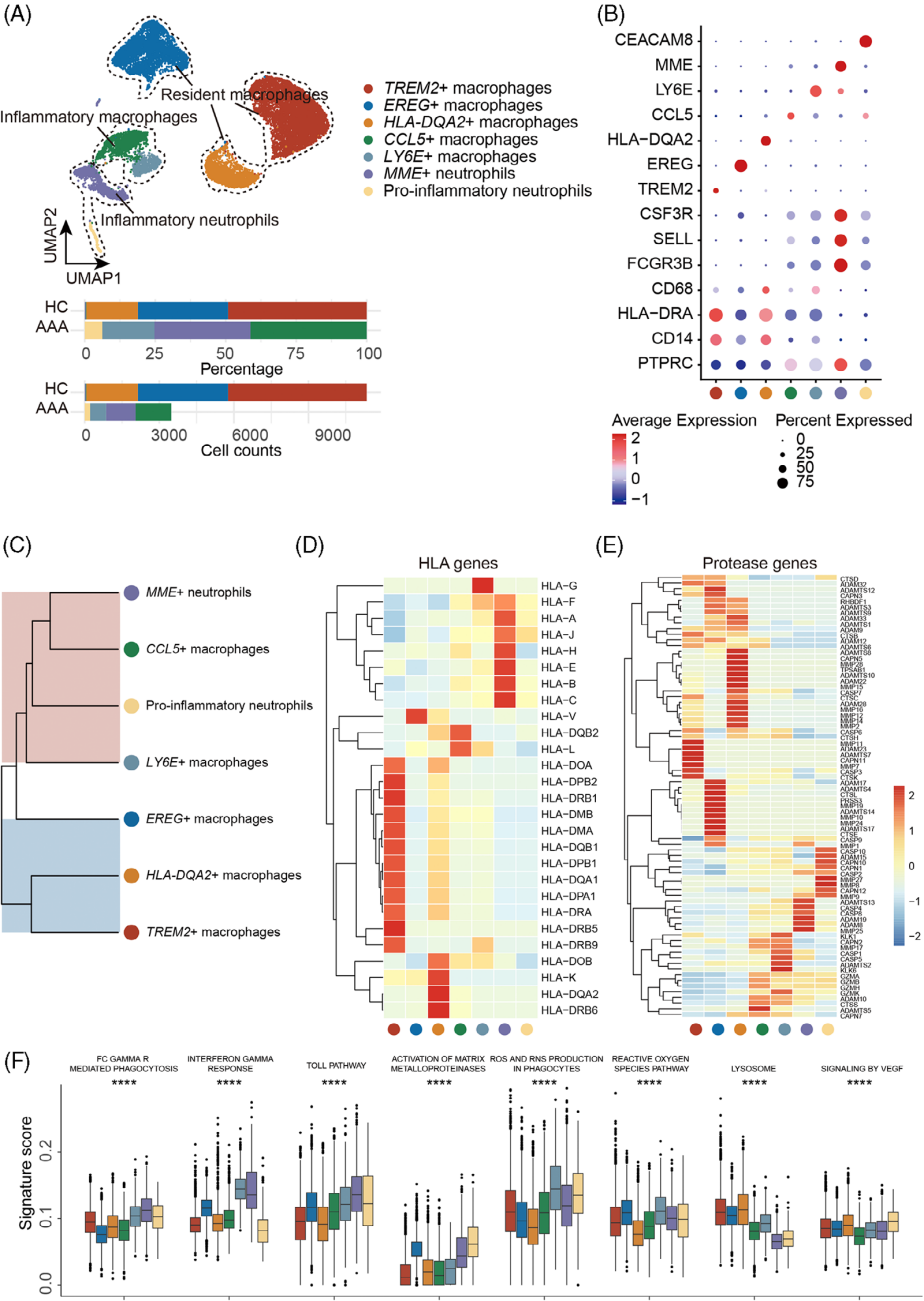
The lineage and characteristics of macrophage and neutrophil subsets between contents of human abdominal aortic aneurysm and healthy aortic tissues. (A) Uniform manifold approximation and projection (UMAP) plot displaying the distribution of macrophage and neutrophil subsets, with a horizontal bar plot indicating the composition and count of these cell types. (B) Bubble plot of marker genes of macrophage and neutrophil subsets. The size of each dot indicates the percentage of expressed genes, and the colour scale bar reflects the average expression. (C) Hierarchical cluster analysis of different macrophage and neutrophil subsets. Colour covering the branches indicates the enrichment in the abdominal aortic aneurysm (AAA) (red) or healthy aorta (blue). (D) Heatmap showing normalized average expression of major histocompatibility complex (MHC)‐associated genes in each macrophage and neutrophil subset. (E) Heatmap showing normalized average expression of protease‐associated genes in each macrophage and neutrophil subset. (F) Boxplot depicting signature score of various phenotypic gene sets regarding macrophages and neutrophils. One‐way analysis of variance (ANOVA) was performed; ****p < .0001.

Dysregulation of protease activity is critical for extracellular matrix remodeling‐a key feature in AAA development and progression. Our analysis revealed the presence of specific protease genes in different populations, with MME+ neutrophils expressing high ADAMT/ADAM (Figure 2E). Additionally, assessment of function‐related gene set scores showed that inflammatory macrophages and MME+ neutrophils displayed a relatively high response to interferon, Toll pathway activation, and reactive oxygen species production, while exhibiting relatively low scores for lysosome and angiogenesis (Figure 2F).

We conducted a pseudotime developmental trajectory analysis of inflammatory neutrophil and macrophage subsets to explore their differentiation relationships. Our results indicate that the trajectory originates from MME+ neutrophils, branching into distinct developmental pathways: pro‐inflammatory neutrophils and inflammatory macrophages occupy separate branches. Notably, the trajectory for inflammatory macrophages distinguishes between CCL5+ and LY6E+ macrophages, suggesting unique differentiation outcomes for these subsets (Figure 3A–E). The infiltration levels, indicated by signature scores, showed significantly higher levels of MME+ neutrophils, CCL5+ macrophages, and LY6E+ macrophages in AAA tissues compared to healthy aorta, with MME+ neutrophils exhibiting particularly pronounced differences, unlike pro‐inflammatory neutrophils, which showed no significant variation (Figure 3F). To further validate the role of MME+ neutrophils in AAA, transcriptional and translational levels of MME in AAA of both human and mouse tissues were conducted, revealing high levels of MME expression within AAA tissues (Figure 4A–F) and co‐localized with neutrophils in AAA (Figure 4G).

**FIGURE 3:**
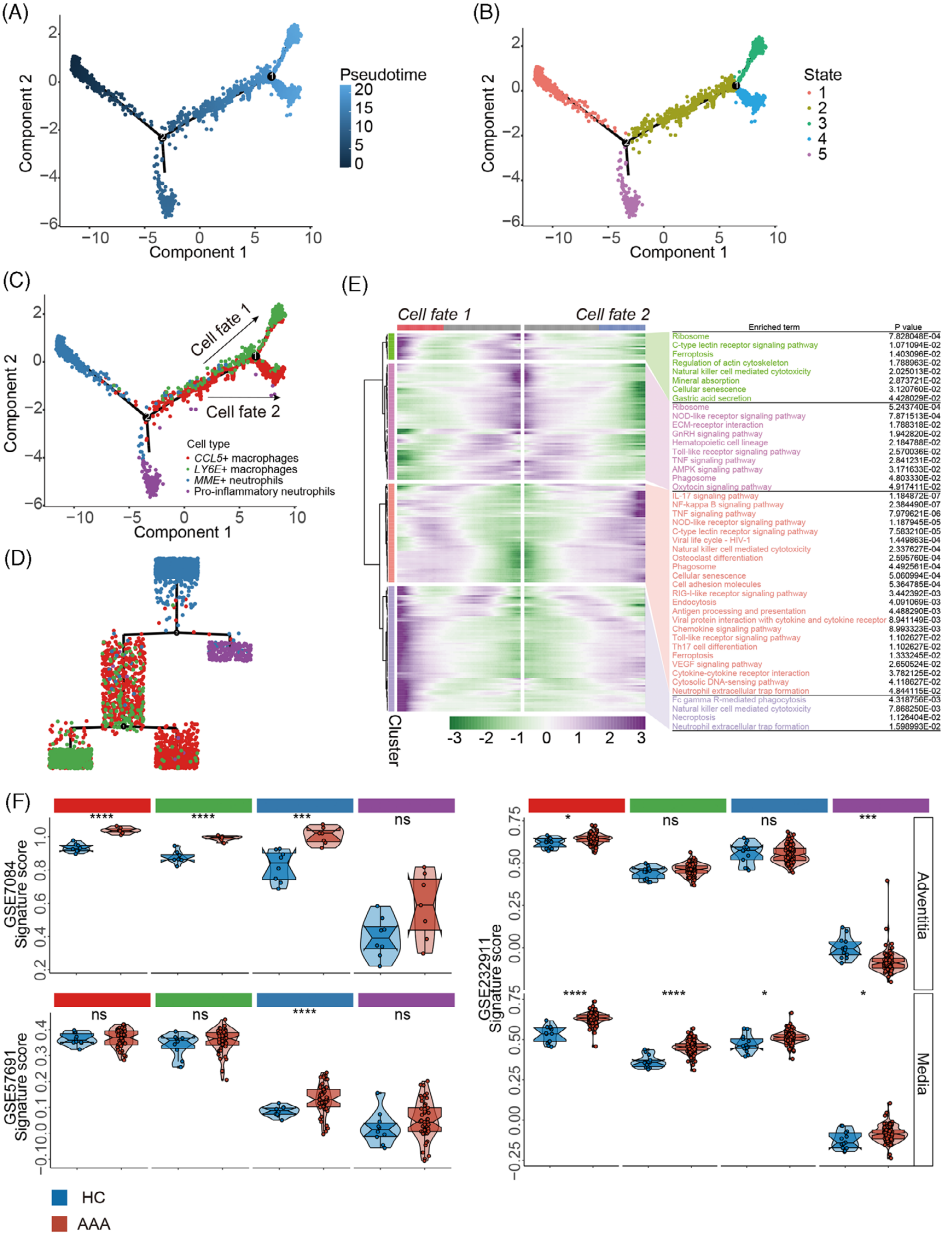
Trajectory analysis of inflammatory neutrophil and macrophage populations. (A) Monocle2 pseudotime trajectory of inflammatory neutrophil and macrophage populations. Cells are colour‐coded based on their pseudotime score, where darker colours indicate early stages and lighter colours represent later stages. (B) Monocle2 pseudotime trajectory of inflammatory neutrophil and macrophage populations with cells coloured by state. (C) Monocle2 pseudotime trajectory of inflammatory neutrophil and macrophage populations with cells coloured by cell types. (D) Hierarchical branch plot showing pseudotime trajectory of inflammatory neutrophil and macrophage populations, with cells coloured to indicate their respective cell types. (E) Heatmap showing different blocks of differentially expressed genes (DEGs) along the pseudotime trajectory (left). Selected Kyoto Encyclopedia of Genes and Genomes (KEGG) pathways related to these DEGs are shown on the right. (F) Violin plot showing the comparison of inflammatory neutrophils and macrophage infiltration between abdominal aortic aneurysm (AAA) and healthy aorta tissues. Non‐paired two‐tailed Student's t‐tests were performed; *p < .05, **p < .01, ****p < .0001 and ns *p* > 0.05.

**FIGURE 4:**
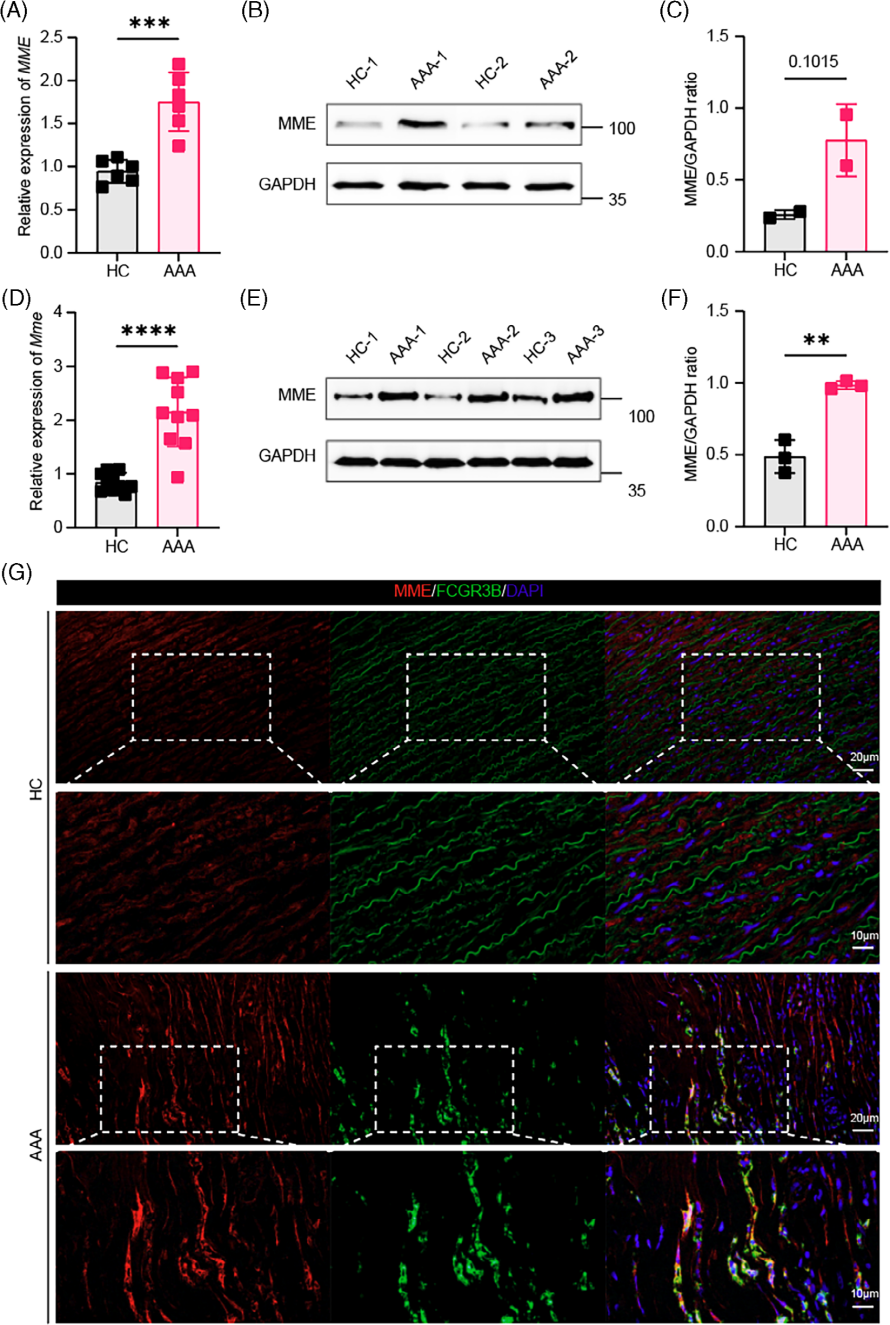
Metalloendopeptidase (MME) is highly expressed and co‐localizes with neutrophils in abdominal aortic aneurysm (AAA) tissues. (A) Quantitative real‐time polymerase chain reaction (qRT‐PCR) analysis reaffirms the elevated transcriptome level of MME in human AAA compared to healthy control. (B, C) Western blot analysis corroborates the abundant translational level of MME in human AAA compared to healthy control. (D) Validation of qRT‐PCR analysis of the heightened transcriptome level of MME in mice AAA. (E, F) Western blot analysis supports the increased translation of MME in mice AAA. (G) The co‐staining experiment suggested highly expressed MME co‐localizes with neutrophil marker FCGR3B within human AAA tissue. Non‐paired two‐tailed Student's t‐tests were performed; **p < .01, ***p < .001 and ****p < .0001.

Gene Set Enrichment Analysis was performed to unravel functional differences between two neutrophil subsets. This analysis revealed that MME+ neutrophils are associated with immune receptor activity, regulation of viral genome replication, apoptotic signalling pathway regulation, neutrophil chemotaxis, and encapsulating structures. These functions underscore the role of MME+ neutrophils in immune modulation, antiviral responses, and structural encapsulation (Figure S2).

To deepen our understanding of the inter‐ and intra‐cellular communication pathways implicated in AAA, we conducted a comprehensive analysis of several enriched pathways. Notably, the chemokine signalling pathway emerged as a critical component across all assessed cell‐cell interactions (Figure S3A). Our analysis uncovered extensive interactions among immune cells within AAA tissues, shedding light on the intricate cellular communication landscape (Figure S3B). Crucially, MME+ neutrophils were identified as key players in this network, serving both as contributors to and recipients of signals. They fulfilled diverse roles as senders, receivers, mediators, and influencers of communication (Figure S3D–F). In a detailed examination of the CXCL signalling pathway molecules within immune cells, we pinpointed CXCL1, CXCL8, CXCR1 and CXCR2 as the primary contributors, further highlighting the significant involvement of MME+ neutrophils in orchestrating inflammatory responses (Figure S3G,H).

In conclusion, our study offers valuable insights into the immune microenvironment of human AAA contents by conducting a thorough assessment of immune cell compositions and their contributions to AAA pathogenesis. Notably, we identify MME+ neutrophils as pivotal inflammatory mediators in the progression of AAA, highlighting their crucial role in driving the inflammatory response within this condition. Future research should focus on further elucidating the mechanisms underlying the activation and function of MME+ neutrophils within the AAA microenvironment, exploring the interplay between neutrophils and other immune cells in AAA. Targeting these specific neutrophils could help mitigate the inflammatory response in AAA, potentially slowing disease progression or even preventing rupture.

## AUTHOR CONTRIBUTIONS

Shuzhen Cheng and Lushun Yuan conceived and designed this study, Shuzhen Cheng and Chuan Liang collected clinical information and biosamples, Lushun Yuan and Shuzhen Cheng performed the analysis procedures, Lushun Yuan analysed the results, Jianliang Zhou contributed analysis tools, Lushun Yuan, Shuzhen Cheng, Chuan Liang and Jianliang Zhou contributed to the writing of the manuscript. Shuzhen Cheng supervised the research. All authors have reviewed the manuscript.

## CONFLICT OF INTEREST STATEMENT

All the authors, including Lushun Yuan, Chuan Liang, Shuzhen Cheng, Jianliang Zhou and Song Chen, hereby confirm that they have no conflict of interest and have nothing to disclose.

## FUNDING INFORMATION

This study is supported by Zhongnan Hospital of Wuhan University (ZNYQ2023002), Young Elite Scientists Sponsorship Program by CAST (grant number: 2022QNRC001) and the National Natural Science Foundation of China (grant number: 82270382). The funders were not involved in the study design, data collection, analysis, publication decisions, or manuscript preparation.

## ETHICS STATEMENT

All use of tissue specimens and clinical information was approved by the Ethics Committee at Zhongnan Hospital of Wuhan University (No. 2022140K), and all research procedures were compliant with the Helsinki Declaration.

## PATIENT CONSENT STATEMENT

Written informed consent was obtained from the patient for publication of this case report and any accompanying images. A copy of the written consent is available for review.

## Supporting information

Supporting Information

Supporting Information

Supporting Information

## Data Availability

The datasets used and/or analysed during the current study are listed as Supplementary data and uploaded to the public database Mendeley Data (doi: 10.17632/84hkcs66h3.1).
