# Supplementary material for: Integration of single‐cell and bulk RNA sequencing unravels metalloendopeptidase+ neutrophils as key inflammatory drivers in abdominal aortic aneurysm
Source: Clin Transl Med. 2024 Dec 2;14(12):e70121. doi: 10.1002/ctm2.70121 (PMC11612259; doi:10.1002/ctm2.70121)
Supplement: Supplementary file 1 — Supporting Information [file CTM2-14-e70121-s003.docx]

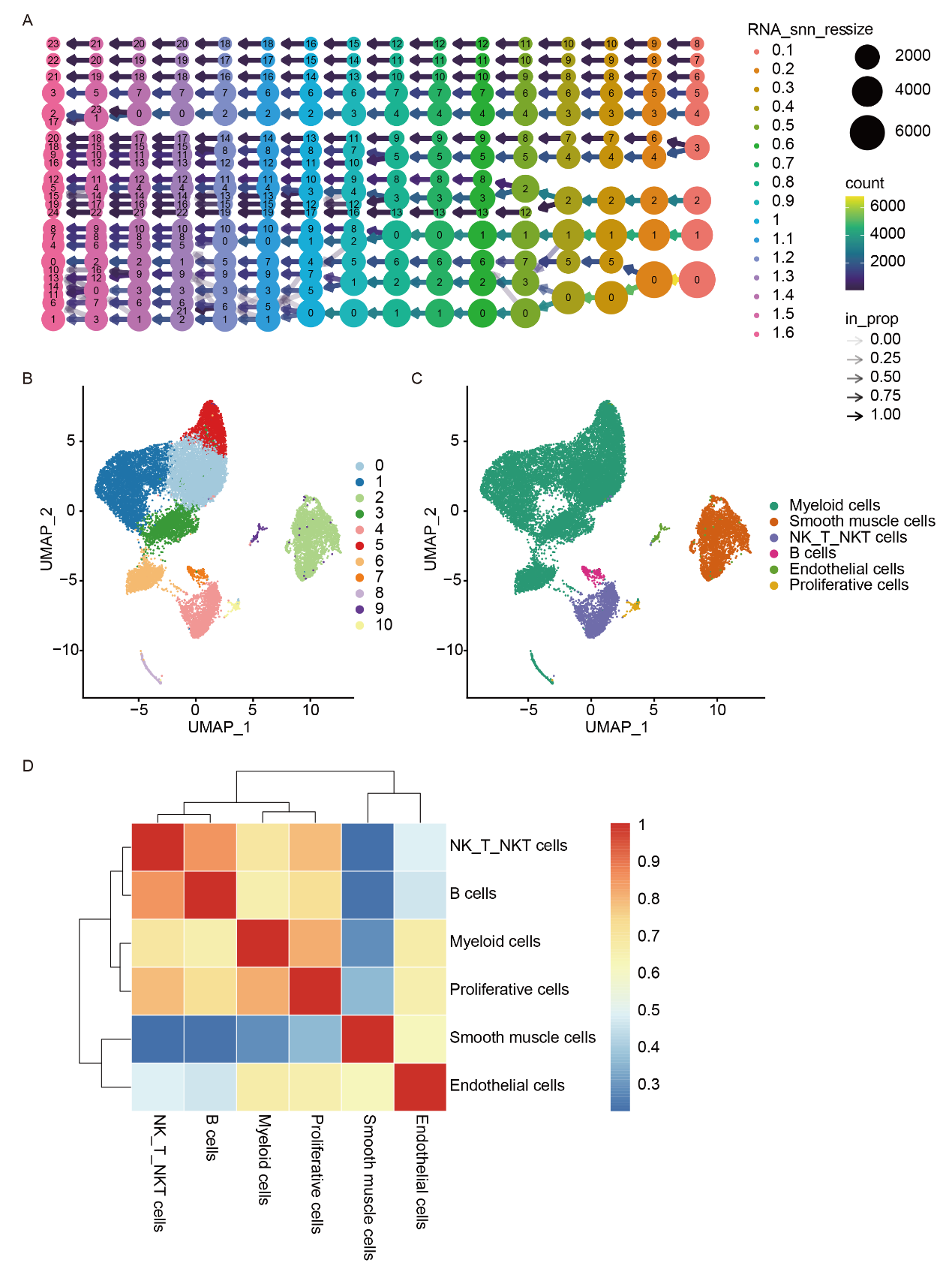


**Supplementary Figure 1. Identification of major cell populations.** (A) Clustering tree showing evaluation for all cell clusters at different resolutions. (B) UMAP plot showing different cell clusters at resolution 0.3, color-coded by different clusters. (C) UMAP plot showing major cell populations, color-coded by five major cell populations. (D) Heatmap showing the correlation between five major cell populations.


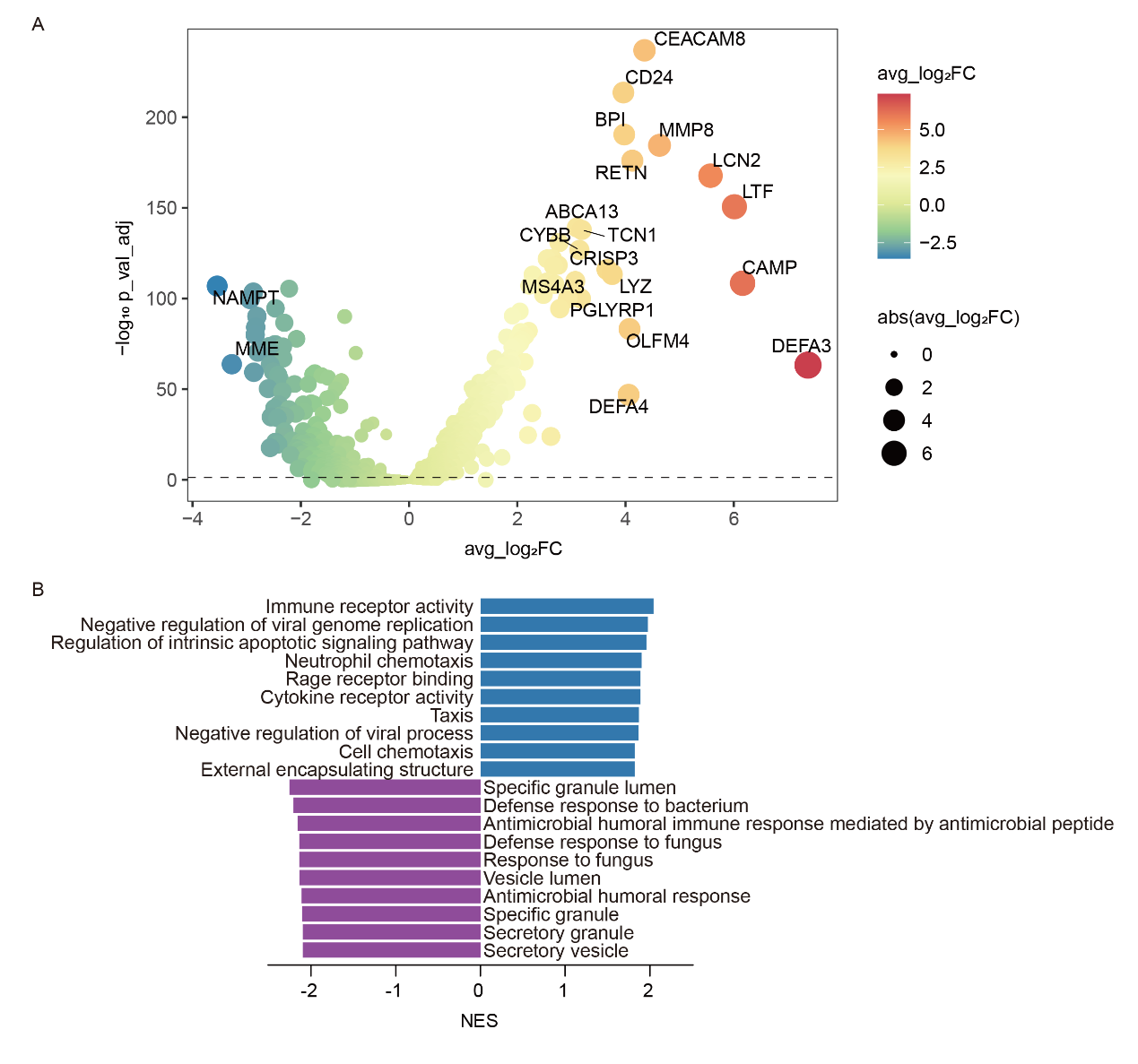


**Supplementary Figure 2. Differentially expressed genes and functions between two inflammatory neutrophils.** (A) Volcano plot showing differentially expressed genes between two inflammatory neutrophils. (B) Gene set enrichment analysis of two inflammatory neutrophils showing top 10 biological process terms.


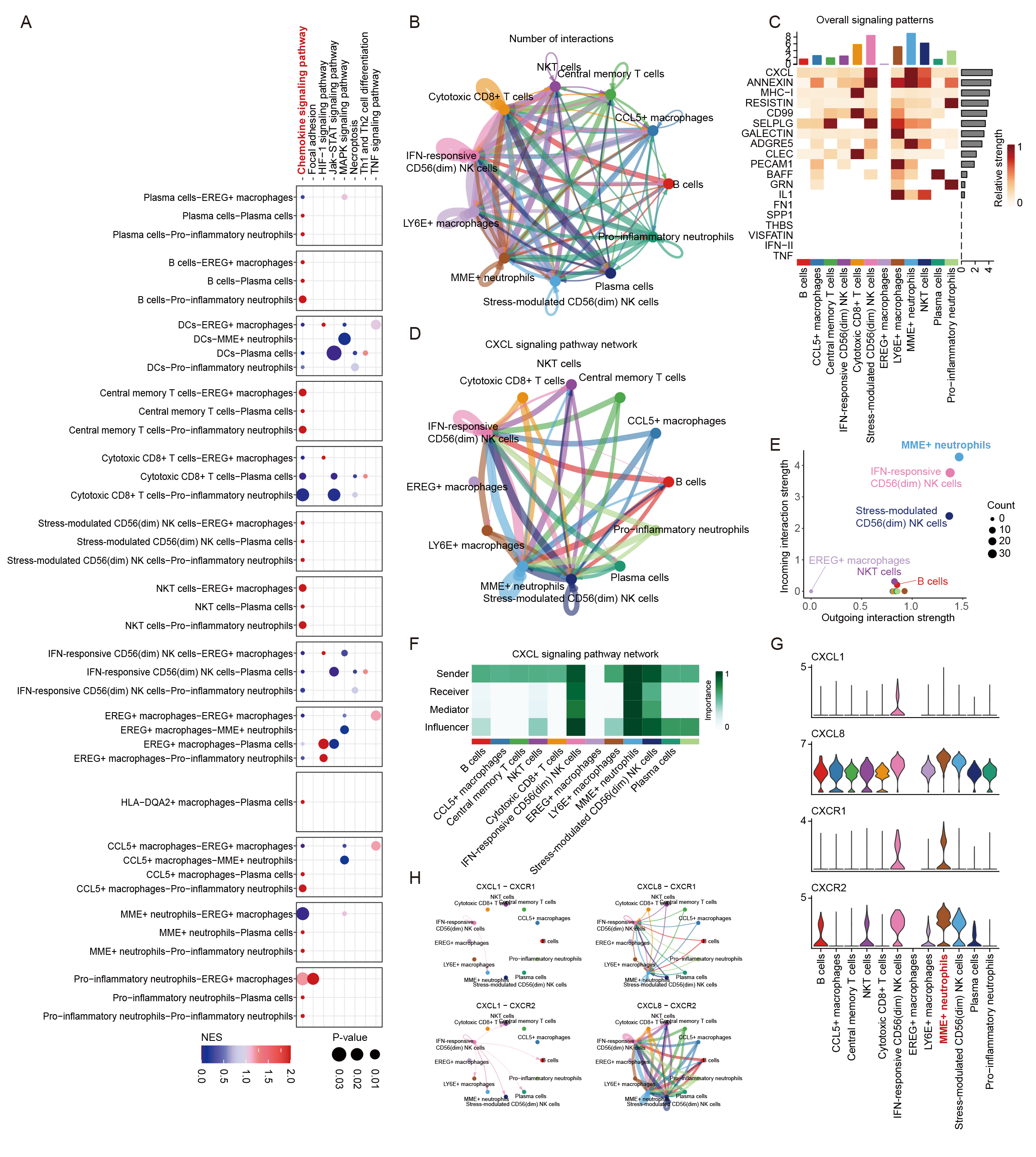


**Supplementary Figure 3. Analysis of cell-cell communications among immune cell populations, with a focus on inflammatory neutrophils and macrophages.** (A) Bubble plot visualizes significantly activated pathways involved in the intercellular crosstalk between immune cells. (B) Circle plot displays the number of interactions between immune cells, with line thickness indicating the count of unique ligand-receptor interactions. Loops represent autocrine circuits. (C) Heatmap depicts the overall signaling patterns of cells within AAA contents. The color reflects the contribution score derived from pattern recognition analysis, where a higher score indicates greater enrichment of the corresponding cell type in the signaling pathway. (D) The inferred CXCL signaling networks presented with edge width representing the probability of communication. (E) Scatter plot showing signaling role analysis on the aggregated cell-cell communication network of CXCL signaling pathway. Size of dot represents the numbers of cell-cell interaction. (F) Heatmap shows the computed centrality scores, identifying the major roles of immune cell groups in the CXCL signaling pathway. (G) Violin plot displays the expression levels of the primary contributors (ligand-receptor pairs) in the CXCL signaling pathway across immune cell groups. H. Circle plot illustrating separated ligand-receptor pairs that regulate cell-cell communication between immune cells.
