# Supplementary material for: Integration of single‐cell and bulk RNA sequencing unravels metalloendopeptidase+ neutrophils as key inflammatory drivers in abdominal aortic aneurysm
Source: Clin Transl Med. 2024 Dec 2;14(12):e70121. doi: 10.1002/ctm2.70121 (PMC11612259; doi:10.1002/ctm2.70121)
Supplement: Supplementary file 2 — Supporting Information [file CTM2-14-e70121-s002.docx]

| **Table S1.** Information of participants (n = 6). | | | | | | |
| --- | --- | --- | --- | --- | --- | --- |
| Variable | AAA1 | AAA2 | AAA3 | Control1 | Control2 | Control3 |
| Sex | Female | Male | Male | Male | Male | Male |
| Ethnicity | Han | Han | Han | Han | Han | Han |
| Race | East Asian | East Asian | East Asian | East Asian | East Asian | East Asian |
| Age (y) | 76 | 60 | 63 | 51 | 33 | 44 |
| Tissue | AAA | AAA | AAA | Healthy aorta | Healthy aorta | Healthy aorta |
| Aortic diameter (cm) | 5.5 | 6.6 | 6.7 | 2.0 | 2.4 | 2.2 |
| Smoking status | Never | Past | Past (quit 2015) | Current | Never | Never |
| Diabetes | No | No | No | No | No | No |
| Hypertension | Yes | Yes | Yes | No | No | No |
| COPD | No | No | Yes | No | No | No |
