# Supplementary material for: Integration of single‐cell and bulk RNA sequencing unravels metalloendopeptidase+ neutrophils as key inflammatory drivers in abdominal aortic aneurysm
Source: Clin Transl Med. 2024 Dec 2;14(12):e70121. doi: 10.1002/ctm2.70121 (PMC11612259; doi:10.1002/ctm2.70121)
Supplement: Supplementary file 3 — Supporting Information [file CTM2-14-e70121-s001.docx]

**Materials and methods**

**Enrollment of study participants and collection of tissue samples**

The study protocol for human tissue sample collection was approved by the Ethics Committee at Zhongnan Hospital of Wuhan University (No. 2022140K). Prior to enrollment, all participants or legally authorized representatives of organ donors provided written informed consent. All experiments involving human tissue samples were conducted in strict compliance with applicable guidelines and regulations. Control aortic samples were procured from individuals who underwent heart transplants, while diseased aortic tissue samples were obtained from patients diagnosed with AAA. Patients with abdominal aortic dissection were excluded from the study.

**scRNA‑seq data preprocessing and analysis**

The raw reads underwent initial processing using fastQC and fastp to eliminate low-quality reads. Removal of poly-A tails and adaptor sequences was carried out using cutadapt. Following quality control, the reads were aligned to the reference genome GRCh38 through the utilization of STAR. The featureCounts software was employed to obtain gene counts and UMI counts. To facilitate further analysis, expression matrix files were created using the obtained gene counts and UMI counts.

The gene-expression matrix was converted into a Seurat object using the Seurat package (v4.4.0) in R (v4.3.1) [1]. To eliminate poor-quality cells, cells with more than 15,000 unique molecular identifiers (UMIs), over 30% mitochondrial gene content, and higher than 1% haemoglobin gene content were discarded. Only cells expressing between 500 and 5000 genes, and genes expressed in at least 3 cells, were retained for subsequent analysis. The DoubletFinder package (v2.0.3) in R [2] was used to further identify and exclude doublet cells from the remaining cells. Following quality control, the integration workflow was performed using the harmony package (v0.1.1) in R [3]. Dimensionality reduction of all cells was conducted based on highly variable genes (HVGs) and the top 20 principal components, determined by an Elbow plot. Graph-based clustering was employed using the "FindNeighbors" function in the Seurat package, with the top 20 principal components. The Clustree package (v0.5.0) was utilized to determine the appropriate resolution, with the "FindClusters" function setting the "resolution" parameter to 0.4. Visualization plots were generated using the two-dimensional uniform manifold approximation and projection (UMAP) model through the "RunUMAP" function, with dimensions set to 1:30. Well-known marker genes were used to assign known cell lineages to the major cell clusters projected in the UMAP model. Subsequently, the major cell clusters were manually assigned to their corresponding cell types based on established markers. The "FindAllMarkers" function (default parameters) was employed to identify markers for all cell populations. For subclustering within major cell populations, the same procedure of identifying HVGs, dimensionality reduction, and clustering was repeated.

**Bulk dataset preprocessing and analysis**

We conducted a thorough search for publicly available gene expression datasets related to AAA. The raw data and clinical information from three specific datasets, namely GSE7084 [4], GSE57691 [5], and GSE232911 [6], were obtained from the Gene Expression Omnibus (GEO) database (<https://www.ncbi.nlm.nih.gov/geo/>). Subsequently, we processed the raw data of the microarray dataset using the robust multi-chip averaging (RMA) algorithm for background correction, log_2_ transformation, quantile normalization, and annotation. These preprocessing steps were performed using the Affy package in R [7]. In cases where multiple probes mapped to a single gene symbol, we selected the probe with the highest expression level for gene annotation.

**Functional‑enrichment analysis**

To explore the functions of different cell types, the FindAllMarkers function was used to investigate the markers of all cell populations. ClusterProfiler (v4.9.3) [8] and fgsea (1.27.1) [9] R packages were used to analyze functional differences in the corresponding cell populations.

**Assessing the signature scores of different phenotypes**

We obtained functional phenotype signatures from the GSEA database (https://www.gsea-msigdb.org/gsea/index.jsp). The M1/M2 phenotype-related signature genes were sourced from Azizi et al. [10]. Genes associated with "classically activated" (M1) macrophages included IL23, TNF, CXCL9, CXCL10, CXCL11, CD86, IL1A, IL1B, IL6, CCL5, IRF5, IRF1, CD40, IDO1, KYNU, CCR7. On the other hand, IL4R, CCL4, CCL13, CCL20, CCL17, CCL18, CCL22, CCL24, LYVE1, VEGFA, VEGFB, VEGFC, VEGFD, EGF, CTSA, CTSB, CTSC, CTSD, TGFB1, TGFB2, TGFB3, MMP14, MMP19, MMP9, CLEC7A, WNT7B, FASL, TNFSF12, TNFSF8, CD276, VTCN1, MSR1, FN1, and IRF4 were used to define the signature of "alternatively activated" (M2) macrophages. To infer phenotype-related scores for selected cell populations, we employed the AUCell package (v1.23.0), utilizing the AUCell algorithm with default settings.

Marker gene sets for immune microenvironment infiltration were derived from our current study (top 30 marker genes) and the studies of Bindea et al. [11] and Charoentong et al. [12]. In this study, the ssGSEA algorithm was employed to assess the relative abundance of infiltrating immune cells in AAA and healthy aorta tissues from bulk datasets.

**Developmental trajectory inference**

To explore the differentiation trajectories of the selected clusters, we utilized the Monocle2 algorithm implemented in the monocle R package (v2.29.0) [13]. To separate the clusters of interest, we employed the "subset" command from Seurat. To construct a CellDataSet object, we utilized the "newCellDataSet" function of monocle2, setting the "lowerDetectionLimit" parameter to 0.5. Subsequently, we filtered out low-quality cells and genes by applying the "detectGenes" function and the "subset" function, respectively. The "min_expr" parameter was set to 0.1 for this filtering step, which was performed after computing size factors and estimating dispersions. To identify differentially expressed genes among clusters along the trajectory, we employed the "differentialGeneTest" function. For dimensionality reduction, we utilized the "reduceDimension" function with the "DDRTree" method. Visualization of cell ordering was conducted using the "plot cell trajectory," "plot genes in pseudotime," and "plot genes branched heatmap" functions.

**Evaluation of metabolic activity at single‑cell resolution**

The metabolic activities of individual cells within each cell population were visualized and quantified using scMetabolism (v0.2.1), a recently established computational pipeline for quantifying metabolic activities in single cells [14]. The KEGG metabolic gene sets were used for analysis in this study, with the method set to “VISION.”

Differential metabolic pathway activity was determined by comparing different cell types by using the package limma in R [15], which estimates metabolic pathway activity changes by implementing an empirical Bayesian approach. The significance criteria for determining DEGs were set as a false discovery rate (FDR) < 0.05.

**Cell–cell‑communication analysis**

To comprehensively describe the cell–cell interactions between immune cells in AAA content, cellcall (v1.0.7) [16] and CellChat (v1.6.1) [17] were used. Cellcall, a versatile toolkit that integrates paired ligand-receptor to infer inter- and intracellular communication pathways. With CellCall's embedded pathway activity analysis, we identified significantly activated pathways involved in intercellular crosstalk between specific cell types. CellChat is a computational method that utilizes manifold learning and quantitative contrasts to classify signaling pathways and identify both shared and context-specific pathways across diverse datasets. We here included two ligand-receptor databases (secreted signaling and ECM-receptor) for further analysis and cell-cell communication networks, signaling patterns contribution, and contribution of ligand-receptor pairs were analyzed.

**Quantitative real-time polymerase chain reaction (qRT-PCR)**

Total RNA extraction from AAA and healthy aorta tissues was performed using the HiPure Total RNA Mini Kit (Cat. #R4111-03, Magen, China). Subsequently, reverse transcription was carried out using the ReverTra Ace qPCR RT Kit (Toyobo, China). QRT-PCR was conducted using iQTM SYBR® Green Supermix from Bio-Rad in the USA. The fold enrichment was determined using the 2^−ΔΔCt^ method and normalized to GAPDH expression.

**Western blot**

AAA and normal aortic tissues lysates and protein samples were prepared with RIPA buffer, protease inhibitor, and phosphatase inhibitor (Sigma‒Aldrich, USA). The Bradford protein assay (Bio-Rad, Germany) was used to evaluate the protein concentration. Western blot analysis was conducted following the fractionation of total protein samples through 7.5-15% SDS‒PAGE. Immunoreactive bands were visualized using an enhanced chemiluminescence kit (Bio-Rad, USA) and were then detected using a Molecular Imager ChemiDoc XRS + Imaging System (Bio-Rad, USA).

**Immunofluorescence staining**

Immunofluorescence staining was conducted on fixed abdominal aortic aneurysm (AAA) tissues by Biofavor Biotech Ltd. in Wuhan, China. ActinRed staining solution was applied to the cell slide at a 1:600 dilution and left to incubate at room temperature for 20 minutes. Following incubation, the slides were washed three times with PBS, sealed with DAPI, and examined using confocal fluorescence microscopy (Nikon C2+ Confocal Microscope, Japan).

**Statistical analyses**

All statistical analyses were conducted using R software (v4.3.1). The images were created using R Studio. Student's t-test and Kruskal-Wallis test were utilized as appropriate. P-values greater than 0.05 were considered non-significant (ns), while p-values less than 0.05 were denoted as follows: *p < 0.05, **p < 0.01, ***p < 0.001, and ****p < 0.0001. For GSEA analysis, the p-values were additionally adjusted based on the false discovery rate (FDR) to account for multiple-hypothesis testing. To evaluate associations between hypoxia score and metabolic pathway activity, as well as functional signature and metabolic pathway activity, Pearson’s correlation analyses were performed.

**Reference**

1. Hao Y, Hao S, Andersen-Nissen E, Mauck WM, 3rd, Zheng S, Butler A et al. Integrated analysis of multimodal single-cell data. Cell 2021; 184(13):3573-3587 e3529.

2. McGinnis CS, Murrow LM, Gartner ZJ DoubletFinder: Doublet Detection in Single-Cell RNA Sequencing Data Using Artificial Nearest Neighbors. Cell Syst 2019; 8(4):329-337 e324.

3. Korsunsky I, Millard N, Fan J, Slowikowski K, Zhang F, Wei K et al. Fast, sensitive and accurate integration of single-cell data with Harmony. Nat Methods 2019; 16(12):1289-1296.

4. Lenk GM, Tromp G, Weinsheimer S, Gatalica Z, Berguer R, Kuivaniemi H Whole genome expression profiling reveals a significant role for immune function in human abdominal aortic aneurysms. BMC Genomics 2007; 8:237.

5. Biros E, Gabel G, Moran CS, Schreurs C, Lindeman JH, Walker PJ et al. Differential gene expression in human abdominal aortic aneurysm and aortic occlusive disease. Oncotarget 2015; 6(15):12984-12996.

6. Lindquist Liljeqvist M, Hultgren R, Bergman O, Villard C, Kronqvist M, Eriksson P et al. Tunica-Specific Transcriptome of Abdominal Aortic Aneurysm and the Effect of Intraluminal Thrombus, Smoking, and Diameter Growth Rate. Arterioscler Thromb Vasc Biol 2020; 40(11):2700-2713.

7. Gautier L, Cope L, Bolstad BM, Irizarry RA affy--analysis of Affymetrix GeneChip data at the probe level. Bioinformatics 2004; 20(3):307-315.

8. Wu T, Hu E, Xu S, Chen M, Guo P, Dai Z et al. clusterProfiler 4.0: A universal enrichment tool for interpreting omics data. Innovation (Camb) 2021; 2(3):100141.

9. Korotkevich G, Sukhov V, Budin N, Shpak B, Artyomov MN, Sergushichev A Fast gene set enrichment analysis. bioRxiv 2019.

10. Azizi E, Carr AJ, Plitas G, Cornish AE, Konopacki C, Prabhakaran S et al. Single-Cell Map of Diverse Immune Phenotypes in the Breast Tumor Microenvironment. Cell 2018; 174(5):1293-1308 e1236.

11. Bindea G, Mlecnik B, Tosolini M, Kirilovsky A, Waldner M, Obenauf AC et al. Spatiotemporal dynamics of intratumoral immune cells reveal the immune landscape in human cancer. Immunity 2013; 39(4):782-795.

12. Charoentong P, Finotello F, Angelova M, Mayer C, Efremova M, Rieder D et al. Pan-cancer Immunogenomic Analyses Reveal Genotype-Immunophenotype Relationships and Predictors of Response to Checkpoint Blockade. Cell Rep 2017; 18(1):248-262.

13. Qiu X, Mao Q, Tang Y, Wang L, Chawla R, Pliner HA et al. Reversed graph embedding resolves complex single-cell trajectories. Nat Methods 2017; 14(10):979-982.

14. Wu Y, Yang S, Ma J, Chen Z, Song G, Rao D et al. Spatiotemporal Immune Landscape of Colorectal Cancer Liver Metastasis at Single-Cell Level. Cancer Discov 2022; 12(1):134-153.

15. Ritchie ME, Phipson B, Wu D, Hu Y, Law CW, Shi W et al. limma powers differential expression analyses for RNA-sequencing and microarray studies. Nucleic Acids Res 2015; 43(7):e47.

16. Zhang Y, Liu T, Hu X, Wang M, Wang J, Zou B et al. CellCall: integrating paired ligand-receptor and transcription factor activities for cell-cell communication. Nucleic Acids Res 2021; 49(15):8520-8534.

17. Jin S, Guerrero-Juarez CF, Zhang L, Chang I, Ramos R, Kuan CH et al. Inference and analysis of cell-cell communication using CellChat. Nat Commun 2021; 12(1):1088.
